# A commentary on ‘Moderate and severe exacerbations have a significant impact on health-related quality of life, utility, and lung function in patients with chronic obstructive pulmonary disease: a meta-analysis’

**DOI:** 10.1097/JS9.0000000000001448

**Published:** 2024-04-11

**Authors:** Xiuli Liu, Linru Song, Yuanyuan Wang, Yanqiu Li

**Affiliations:** a Department of Pulmonary and Critical Care Medicine, Yantai Yuhuangding Hospital; b Department of Respiratory Intensive Care Unit, Yantai Yuhuangding Hospital, Yantai, Shandong, People’s Republic of China

*Dear Editor,*

It was with great interest that we read the article published in the International Journal of Surgery. Guo *et al*.^1^ conducted a meta-analysis on patients with chronic obstructive pulmonary disease (COPD). Research indicates that moderate and severe exacerbations affect patients with COPD’s utility, lung function, and health-related quality of life (HRQoL) significantly and over time. This emphasizes the need for exacerbation prevention strategies that work.

COPD is a heterogeneous condition with a complex progression of many clinical presentations and deteriorates over time, which vary in both their presence and severity^2^. The lungs’ reduced ability to function, a decline in life satisfaction, and an increase in abrupt flare-ups of the illness are all linked to COPD getting worse. Clinical management and long-term professional care were found to lower the incidence of COPD exacerbations in the future, according to certain studies. For older patients with COPD, the continuous nursing intervention method combined with breathing training guidance has a significant impact on improving the patients’ rehabilitation effect. The patient’s quality of life, self-care skills, and pulmonary function markers can all be significantly enhanced by the continuous nurse intervention method. Additionally, it lessens the negative feelings that lead to a patient’s lack of subjective well-being and effectively enhances their capacity for self-care. According to the Global Initiative for Chronic Obstructive Lung Disease (GOLD) report, treating COPD with a personalized approach is crucial, with the objectives being minimizing disease activity, preventing disease progression, and lowering the risk of recurrent exacerbations.

A composite endpoint called clinically meaningful deterioration (CID) is used to evaluate the intricate course of COPD in a comprehensive manner^3^. When the criteria for a CID were first established, they included a decline in forced expiratory volume in 1 s (FEV1) of at least 100 ml from baseline, an increase in the total score of the St. George’s Respiratory Questionnaire (SGRQ) of at least 4 units from baseline, and/or the occurrence of a moderate-to-severe exacerbation of COPD (AECOPD). An sudden worsening of COPD symptoms that necessitates the use of extra treatment is known as AECOPD. The established minimum clinically relevant difference (MCID) for these outcomes was determined to be the FEV1 and SGRQ thresholds. This endpoint has been adopted as a post-hoc measure in several short-term (6-month to 12-month) COPD studies, and correlations between short-term CID and long-term results have also been documented.

The purpose of this study is to examine how patients’ utility, HRQoL, and lung function are affected by moderate and severe COPD exacerbations. This innovative method offers wise counsel and motivation for this subject of study’s continued advancement. There were a few restrictions, though. First, some of the studies were post-hoc assessments of RCTs, while others were prospective RCTs that assessed the composite outcome^4^. Second, because the SGRQ was only evaluated annually, it was not feasible to evaluate the COPD patients’ conditions more quickly. The SGRQ is complicated and challenging to evaluate frequently in clinical practice, though, and some research indicates that evaluating COPD patients’ conditions annually may also be crucial in predicting long-term clinical outcomes down the road^5^. Finally, the analysis may have been constrained by the narrow age range.

Future clinical trials should be more robust in order to obtain more exact results. The association between the long-term results of upcoming clinical trials and the health of people with COPD thresholds may require more investigation. Undoubtedly, additional research aimed at precisely evaluating the influence of aging on the state of COPD patients is required to validate these results.

## Ethical approval

Not applicable.

## Consent

Not applicable.

## Author contribution

X.L. and L.S.: study concept or design, data collection, data analysis or interpretation, writing the paper; Y.W.: data collection; Y.L.: study concept or design, writing, and revise the paper. X.L. and L.S. equally contributed to the article.

## Conflicts of interest disclosure

There are no conflicts of interest.

## Research registration unique identifying number (UIN)

Not applicable.

## Guarantor

Yanqiu Li.

## Data availability statement

Not applicable.

## Provenance and peer review

This manuscript is a comment without being invited.
